# Cultural Adaptations of Prolonged Exposure Therapy for Treatment and Prevention of Posttraumatic Stress Disorder in African Americans

**DOI:** 10.3390/bs4020102

**Published:** 2014-05-14

**Authors:** Monnica T. Williams, Emily Malcoun, Broderick A. Sawyer, Darlene M. Davis, Leyla Bahojb Nouri, Simone Leavell Bruce

**Affiliations:** 1 Department of Psychological and Brain Sciences, Center for Mental Health Disparities, University of Louisville, Louisville, KY 40292, USA; E-Mails: broderick.sawyer@louisville.edu (B.A.S.); darlene.davis@louisville.edu (D.M.D.); lvbaho01@louisville.edu (L.B.N.); simone.leavell@louisville.edu (S.L.B.); 2 Department of Visual Studies, University of Pennsylvania, Philadelphia, PA 19104, USA; E-Mail: emalcoun@mail.med.upenn.edu

**Keywords:** posttraumatic stress disorder, trauma, African Americans, prolonged exposure, racism, therapeutic alliance

## Abstract

Posttraumatic stress disorder (PTSD) is a highly disabling disorder, afflicting African Americans at disproportionately higher rates than the general population. When receiving treatment, African Americans may feel differently towards a European American clinician due to cultural mistrust. Furthermore, racism and discrimination experienced before or during the traumatic event may compound posttrauma reactions, impacting the severity of symptoms. Failure to adapt treatment approaches to encompass cultural differences and racism-related traumas may decrease treatment success for African American clients. Cognitive behavioral treatment approaches are highly effective, and Prolonged Exposure (PE) in particular has the most empirical support for the treatment of PTSD. This article discusses culturally-informed adaptations of PE that incorporates race-related trauma themes specific to the Black experience. These include adding more sessions at the front end to better establish rapport, asking directly about race-related themes during the assessment process, and deliberately bringing to the forefront race-related experiences and discrimination during treatment when indicated. Guidelines for assessment and the development of appropriate exposures are provided. Case examples are presented demonstrating adaptation of PE for a survivor of race-related trauma and for a woman who developed internalized racism following a sexual assault. Both individuals experienced improvement in their posttrauma reactions using culturally-informed adaptations to PE.

## 1. Introduction

### 1.1. Posttraumatic Stress Disorder

Posttraumatic stress disorder (PTSD) is a highly disabling disorder characterized by re-experiencing, avoidance, hyperarousal symptoms, and cognitive disturbances resulting from experiencing or witnessing an extremely frightening or life-threatening event [1]. Though first acknowledged in combat veterans and rape victims, other traumatic experiences, such as industrial accidents, terrorist attacks, and natural disasters, also have the potential to produce PTSD symptoms [2,3]. The vast majority of individuals meeting criteria for PTSD also meet criteria for at least one other psychiatric disorder [4], with a substantial percentage meeting criteria for three or more other psychiatric diagnoses (59% of men and 44% of women) [4]. This article focuses on race-related trauma, which has previously been under-recognized and under-treated [5,6].

Changes made to the PTSD criterion in the Diagnostic and Statistical Manual-5 (DSM-5) [1] may be a significant step toward multicultural considerations in diagnosis. The definition of a trauma has been broadened to encompass learning about a traumatic event involving a close friend or family member, or repeated exposure to details about a trauma [1]. This may be particularly relevant in the diagnosis of PTSD in African Americans who may be traumatized by familial, historical, or sociopolitical accounts of discrimination or ethnoviolence [5]. While this change to the DSM illustrates the potential for considering discrimination as a particular traumatic event, the current DSM-5 is limited as it does not yet fully acknowledge it as a potential trauma.

PTSD criteria, previously a three-factor model, now includes four factors to better encompass the disorder and distinguish symptoms from similar disorders such as acute stress disorder [1,7]. To more clearly delineate what constitutes PTSD, the DSM-5 expanded the diagnostic criteria to include persistent blame of self or others, persistent negative emotional state, and reckless behavior. The PTSD subtypes (acute and chronic) have been removed and PTSD has been reconceptualized as a spectrum with two new subtypes. The first new subtype refers to PTSD in children younger than six years of age, and the second refers to those with PTSD who exhibit dissociative symptoms [1].

### 1.2. PTSD in African Americans

#### 1.2.1. Prevalence

PTSD prevalence differs by race and ethnicity. Findings from the National Comorbidity Survey-Replication (NCS-R) and National Survey of American Life (NSAL) indicate that, while African Americans have a lower risk for many anxiety disorders, they have a 9.1% prevalence rate for PTSD, compared to 6.8% in European Americans [8]. African Americans with PTSD also displayed significantly more impairment than traumatized European Americans in productivity and out of role domains, indicating greater difficulty carrying out daily activities [8].

Note that in this article, the term “African American” reflects the ethnic experience associated with darker-skinned Americans of African descent. “Black” is used to describe the ethnic group of darker-skinned individuals with ancestral origins in Africa who may or may not have been born in the U.S. (*i.e.*, Caribbean Black Americans) [9]. It is often assumed that brown-skinned individuals are all “African American” when in fact there are a multitude of races that have darker complexions. Additionally, “White” is used to describe the ethnic group of light-skinned individuals residing in the U.S. with ancestral origins in Europe, or light-skinned individuals still residing in Europe. “European American” reflects such individuals residing in the U.S. with European ancestry.

#### 1.2.2. Trauma Cognitions

PTSD alters perceptions of self, others, and overall safety in the world; cognitions concerning these constructs may differ across racial groups. For example, African Americans with PTSD have been found to have lower expectations about the benevolence of the world than European Americans. When comparing African Americans and European Americans, Zoellner and colleagues [10] reported that African Americans held more negative perceptions of the world, appearing more skeptical and mistrustful. Experiencing a traumatic event changed perceptions of the world in European Americans from positive to negative, yet African American perceptions were not impacted by traumatic experiences [10]. Poulin and Silver [11] examined how age, mental health history, prior experience with stressful life events and other important elements impact one’s belief in the benevolence of the world. Stressful life events were coded and measured based on several surveys aimed at diagnosing possible PTSD. Events were divided into three subgroups: total number of events, bereavement-related events and non-bereavement-related events. The authors found that non-White ethnic groups that have experienced above average rates of recent negative events were more likely to have lower levels of benevolent beliefs than their European American counterparts.

A study of adults with comorbid PTSD and alcohol dependence suggested that African Americans, when compared with European Americans, endorsed stronger negative cognitions about the safety of the world, which the authors attributed to the impact of racism and discrimination [12]. In addition, for African Americans, negative cognitions about the self were correlated with maladaptive drinking behaviors that affected all domains of life, while this relationship was not found in European American subjects. These studies highlight cultural differences found concerning negative cognitions for individuals with PTSD, suggesting that African Americans may view the world more negatively than European Americans.

#### 1.2.3. Treatment Seeking and Barriers to Treatment

The NCS-R results indicated that individuals with PTSD had an increased risk of high school and college failure, teenage pregnancy, marital instability, and unemployment when compared to individuals without PTSD [13]. Given the greater dysfunction experienced by those with PTSD, gaining access to treatment is therefore expected to be more difficult. Obstacles to obtaining treatment for low-income African Americans with PTSD may entail access to transportation, cost of services, family disapproval, and unfamiliarity with processes for receiving treatment [14]. African Americans also have greater feelings of stigma, more negative attitudes concerning mental health treatment, and greater fears of being discriminated against by mental health providers [15,16], which reduces help-seeking.

Lester and colleagues [17] found that African Americans were less likely to complete treatment for PTSD when compared to European Americans. African Americans were 1.5-times more likely to drop out and 3-times more likely not to initiate therapy, despite being more hopeful about treatment benefits prior to therapy. Since they progressed similarly to their European American counterparts before dropping out, even after controlling for demographic variables such as income and education levels, lack of treatment benefit does not explain their higher attrition. The study authors attribute these findings to potentially faster improvement among African Americans, stigma surrounding treatment, and lack of cultural sensitivity in the assessment and treatment process. The latter two attributions are more likely, given the trends outlined in treatment seeking behaviors of African Americans.

#### 1.2.4. Race-Based Trauma

Several studies have established a link between racism and mental health outcomes, including depression [18], substance use [19], and overall psychological distress [20,21]. Although this link has been consistently demonstrated, it is difficult to identify specific aspects of racism that produce the most negative outcomes, given that individuals vary in their reactivity to stress and perceptions of racism. Tools for assessing race-based stress in relation to trauma are needed to assist mental health professionals who may overlook racism and mistakenly attribute psychopathology to other factors [22]. Mental health professionals may not be aware of the effects of racism on psychopathology because of a societal tendency to deny or rationalize the presence of racism [6,23].

Racism-related traumatic experiences can range from frequent ambiguous microaggressions to blatant hate crimes and physical assault. Racial microaggressions are subtle, yet pervasive acts of racial discrimination perpetuated against African Americans and other groups [24,25,26]. These may be brief remarks, vague insults, or non-verbal exchanges [23,25]. When experiencing microaggressions, African Americans lose vital mental energy attempting to pinpoint the intention of one who commits the microaggression [24,25,26]. The more severe race-based stressors, such as physical assault, may be life-threatening, directly causing PTSD. Chronic fear of everyday racial stressors may induce constant vigilance or paranoia, which over time may result in PTSD symptoms, or contribute to PTSD when a more stressful event occurs [6]. In fact, one study of female veterans found that African Americans scored higher on measures of ideas of persecution and paranoia, which the authors attributed to an adaptive response to racism [27].

### 1.3. Psychotherapeutic Treatments for PTSD

Several cognitive behavioral therapies have gained empirical support as effective treatments for PTSD including cognitive processing therapy, cognitive therapy, stress inoculation therapy, and prolonged exposure (PE) [2,28,29,30]. Among these, PE may be particularly effective in the treatment of PTSD [31,32]. PE was developed from emotional processing theory, which poses that unhelpful cognitions resulting from PTSD represent fear structures that produce associations that generalize to but do not accurately predict real life situations. PE corrects erroneous associations in clients’ fear structure, such as perceiving that the world is generally dangerous and that clients are unable to cope with stress [33]. This allows PTSD sufferers to view the trauma event as one time point in the past, and not a continuing concern for all times. In addition to seminal PE research conducted by Foa *et al.* [33], Marshall and Suh [34] developed a training and treatment center for trauma-related psychiatric disorders, where the use of PE was effective in treating PTSD in a multicultural community. Additionally, among a clinical sample of predominately African American women with PTSD, PE was more effective in reducing PTSD and anxiety symptoms compared to other forms of treatment [31]. 

While traumatic events alone are stress-inducing, for African Americans, their history of marginalization, more negative views of the world, and exposure to discriminatory experiences may increase skepticism surrounding mental health treatment [35]. Mental health and other medical practitioners may perpetuate substandard health outcomes through failure to assess historical perspectives potentially influencing patient mistrust of healthcare [36]. This may help explain why African Americans are less likely to see a reduction in symptoms even when participating in empirically supported therapies [37]. African American mistrust of psychotherapy may interfere with treatment outcomes in ways that majority group members do not experience [38].

An individual’s ethnic identity can vary from not identifying with any particular ethnic group, to identifying heavily with one particular group. Ethnic identity development typically begins in adolescence and becomes fully developed in young adulthood. When considering race-related traumas, level of identification with one’s cultural group may alter perception of a racist event [39]. For example, those identifying strongly with their ethnic group may be more attentive to situations where racism may present, which may in turn impact their stress levels. Levels of ethnic identity can also impact therapy, since an African American identifying more strongly with their ethnic group may feel reticent to discuss their race-based trauma with a non-Black clinician, and this also depends on the client’s stage of ethnic identity development [40].

Considering these factors, therapists who are not African Americans may need to address the cultural mistrust African American clients may feel toward them [22] and explicitly initiate assessment and discussion of their client’s race-based trauma. Given the sensitive nature of race-based traumatic experiences, African American clients need to feel very comfortable with their therapists before committing to PE therapy. For this reason, we have adapted the PE protocol to address issues that clinicians may encounter with African American clients. These include potential difficulties building the therapeutic alliance, assessment of the role of racial issues in the trauma, and how to address racial themes during treatment.

## 2. Prolonged Exposure Therapy for African Americans

### 2.1. Cultural Education

Prior to working with any cultural group, the therapists should possess a basic level of cultural knowledge and sensitivity, through formal education or prior meaningful experiences with members of the target group. Cultural understanding is essential to prevent misattribution of symptoms to stereotypes about the client’s race or culture [9,41], such as aggressive reactivity or avoidance of strangers [5]. When psychotherapists appropriately share their own cultural values and acknowledge their client’s cultural background, therapists are perceived as being empathic, so clients are more likely to be more open in therapy [42]. Some therapists believe the best strategy is a colorblind approach, which will tend to ignore issues related to race and culture. Colorblind ideology minimizes ethnic and racial differences and emphasizes commonalities; however, this approach is associated with more negative outcomes [43]. Willingness to engage in an ethnically sensitive approach is therefore essential.

### 2.2. Assessment

Formal assessment of the trauma symptoms is an important initial step prior to the provision of PE. The Posttraumatic Diagnostic Scale (PDS) [44,45], a 49-item measure used in clinical and research settings to evaluate PTSD in relation to an index trauma, is recommended for the initial assessment of PTSD symptoms. The PDS demonstrates strong reliability in African American samples (α = 0.87) [46] and was recently cross-culturally validated in a primarily African American sample [47]. Since African American clients may hesitate to volunteer incidents about racism, especially to European American clinicians [6,48] we recommend that assessors inquire about race-based trauma when completing the trauma checklist (Part 1 of the PDS).

The Standardized Trauma Interview (STI) [49,50] is administered during PE session 1, and is useful for gathering specific information about the trauma and related cognitive and physiological symptoms. Although no validation studies using all African American samples have been conducted to date, the STI has been used to assess a clinical sample comprised of 34% African American participants [49]. As with the PDS, the STI was not designed to include racial issues, but it may be adapted to include instances of racism-related trauma. For example, when assessing attributions of blame and responsibility, the clinician can inquire about whether or not during, prior to, or after their trauma, the client believed race was a factor. Note, however that trauma should be initially identified in the PDS, and the STI may be used to identify race-based themes for processing with imaginal exposure. If a racism-based incident is identified as the index trauma, then it would be the focus of the STI assessment. In such cases, clinicians are encouraged to also assess for racism on the STI item asking about prior “similar” traumas and attributions of blame. This could be particularly relevant since prior experiences of racism may sensitize individuals to PTSD from any other type of subsequent trauma, and not just to trauma by racism [6,48,49,50,51].

To make appropriate cultural adaptations to treatment, the therapist must first understand the client’s culture. African Americans are not culturally homogenous; therefore a cultural assessment is important. Factors particularly relevant for African Americans may include ethnic identity, racism-related stress, and religious beliefs [12,52,53]. To assess these specific constructs therapists could administer the corresponding self-report measures: the Multidimensional Inventory of Black Identity (α = 0.61–0.77 for African Americans) [54], the Multigroup Ethnic Identity Measure (MEIM) (α = 0.76–0.90) [55,56,57], the Schedule of Racist Events (SRE) (α = 0.94–0.95) [58,59], and the Brief Religious Coping (Brief-RCOPE) (subscales: α = 0.87 and 0.78) [60,61] scale. Reviewing the results of such measures can be a good way of starting a discussion about experiences surrounding race and culture. As strong religious beliefs may be more prevalent in African American clients [52,53,62], it may be particularly useful to assess these beliefs, as opposed to generalizing them to all African Americans. Thus, these measures could be used as a means to better understand the client’s values, and the role of faith and culture in their development. For a broader assessment, clinicians might also consider the 16-item DSM-5 Cultural Formulation Interview (CFI) [1].

Self-report measures are regularly administered to measure change in PTSD and depressive symptoms. These measures may typically include Part 2 of the PDS, and the Beck Depression Inventory-II [63], both of which display sound psychometric properties in African American samples [12,64,65].

### 2.3. Psychoeducation and Preparation for Treatment

The first session of PE involves assessment and psychoeducation [66]. (This article assumes a basic familiarity with PE; please see Foa *et al.* [66] for details on the administration PE). The therapist explains the initial “pre-treatment” assessment, which includes the adapted PDS and other cultural measures mentioned above, then discusses the administration of the STI and weekly administration of PDS and BDI-II as “within treatment” assessment. This accomplishes two goals: (1) it helps the clinician plan treatment by identifying the boundaries of imaginal exposure and, if relevant, racism-related cognitions as targets for processing, and (2) tracks treatment progress. After discussing assessment procedures, the therapist then explains the focus of PE treatment, and how it will address particular fears and coping difficulties. To do this, first the therapist will explain how avoidance of certain situations, people, thoughts or feelings may maintain fears, and how PE will enable the client to confront fears (decreasing avoidance) to overcome the trauma. Then the processes of imaginal and *in vivo* exposure are explained to help the client understand how each component will assist them in recovery. In addition to the exposure components of PE, the roles of maladaptive cognitions concerning the trauma are explained. The therapist ensures that the client understands how these cognitions can alter their perception of the trauma, and how changing these negative thoughts or beliefs may help isolate the traumatic event thereby reducing generalization of the fears associated with the trauma.

When treating African American clients, it may be useful to address how racism can be a factor in the development of PTSD (e.g., presenting data and other literature on the mental health consequences of racism and discrimination). In demonstrating openness to understanding the negative feelings associated with being discriminated against, providing psychoeducation of this kind will boost rapport and increase the client’s comfort level with addressing difficult issues in PE therapy. Acknowledgement of the possibility of race-related trauma alone can address experiences salient to minority populations and provide additional validation for clients. However, while it is important to assess for and acknowledge the possibility of race-related trauma, clinicians must not assume all African Americans consider themselves a victim of racism and it may not be relevant to their index trauma.

Empirically-supported treatment approaches for many disorders have not been validated in African American samples [2,67], and African American clients may wonder if PE will be useful for them specifically. Therapists can help address this by discussing ways in which PE has been effective for other African American clients with PTSD. After acknowledging numerous traumatic experiences in different populations due to race, an appropriate transition may be to address how these varying experiences are integrated into PE treatment. This will give clients confidence in the efficacy of PE treatment, increasing their comfort level that their unique experiences, including race-related trauma, will be properly addressed in therapy.

As discussed earlier, PE requires that the client trust the therapist enough to fully share their traumatic experience—an event that is typically characterized by feelings of shame and failure. Many African Americans put considerable effort into maintaining an image of strength (*i.e.*, the “Strong Black Woman” persona [68] and John Henryism [69]) as a defense mechanism to deal with racism and microaggressions. These images involve African American men and woman’s inherent and/or learned strength and resiliency in the face of adversity. As such, both images can be used as positive references to define ideal African American women/men. However, given the nature of PTSD treatment, these strong personas may not be conducive to disclosure of traumatic experience or the associated negative emotions. Therefore, spending considerable time early in the treatment process to develop an authentic connection will facilitate disclosure and help keep the client engaged when treatment gets more difficult (e.g., imaginal exposure). Extra sessions prior to starting exposures should specifically focus on strengthening the therapeutic alliance, overcoming mistrust, and building rapport. Additionally, it is helpful to address any racial issues between therapist and client if they are present. African American clients may be uncomfortable with obvious differences in culture being overlooked and may not directly express this, but it may present as treatment resistance. Ignoring cultural differences can damage the therapeutic alliance if not addressed [43,48]. African Americans’ perception of the role of the therapist may also be different from other ethnic groups, as they see the therapist as more of a vehicle for change (*i.e.*, one who can facilitate change) [22], but not necessarily an agent of change (*i.e.*, one who has absolute power to make changes) [22]. African Americans may be more likely to endorse a stronger belief in their ability to overcome stress through use of personal strengths, which may discourage them from seeking treatment or making full use of it [69]. In addition to cultural differences, gender differences may exist, as males may need more time to become comfortable sharing with therapists compared to females [22].

African Americans are more likely to function in an extended family structure [70,71], as such, utilizing support networks during treatment may improve therapeutic outcomes (see [72]). Having a discussion prior to PE treatment about who among their social network (e.g., family, close friends, church, women’s group, God, *etc.*) will be a safe source of support and strength throughout the treatment process may be extremely beneficial to the client. Completing PE therapy can be very stressful, and encouraging clients to seek out support from others outside of therapy may improve the clients’ ability to commit to PE. Furthermore, clinicians may want to adopt an Afrocentric approach to therapy with African Americans. An Afrocentric approach emphasizes resiliency, optimism, faith, and family support, and these views are prominent in the African American community, playing an important role in psychological functioning [73,74,75,76,77]. However, as previously noted, understanding a client’s value system is imperative before adopting any of these perspectives during psychotherapy. There is considerable heterogeneity within the African American community, and generalizing these principles to all African American clients may be offensive, thereby damaging the therapeutic alliance.

### 2.4. In Vivo Exposure

The *in vivo* exposure component initiates contact between clients and stimuli from their environments that serve as reminders of the trauma. This hierarchy of feared stimuli consists of people, situations, and events that clients avoid to temporarily alleviate anxiety. By participating in increasingly anxiety provoking exposures, clients gain confidence in their abilities to confront feared stimuli, and by doing so rid themselves of various unhelpful thoughts that have formed in relation to these stimuli.

For PE treatment for PTSD, such exposures typically take place outside of session because many objects and situations that clients avoid are not easily accessible in the office setting [78]. However, when clients have difficulty completing exposures independently, it becomes necessary that the therapist directly assist in the exposure process, and this may need to happen outside of the office setting. By completing *in vivo* exposures the client begins to see the process of natural recovery. After completing assigned exposures, it becomes evident to the client that confronting feared stimuli helps to correct maladaptive thoughts that have been erroneously attached to particular stimuli, and as a result anxiety decreases.

When conducting *in vivo* exposures for African Americans that have experienced race-related trauma or racism, it is imperative to assign activities that incorporate these situations, which underscores the need for collaboratively constructing a hierarchy that fully encompasses a client’s race-based fears. The client may have more firsthand knowledge of their race-related triggers which should be utilized during treatment, which may be connected to ubiquitous racism during shopping experiences in the community, on the job in leadership positions, or in educational/learning environments [79]. While typical *in vivo* exposures may include going to feared places or situations, exposures for traumas entailing racism may require interacting with individuals from particular racial or ethnic backgrounds. For example, if a client’s fears have generalized to include all European American males, *in vivo* exposures may include sitting next to a European American man on the subway and starting a conversation. Through repeated exposures the client will learn that most European American males are not dangerous.

Prior to the exposure, it would be helpful to discuss with the client how to conduct such exposures in a safe manner, to minimize the chances of re-experiencing racism. The goal is not to have clients become habituated to racism, but to tolerate their personal emotional response to the possibility of a racist response being made by a person they may engage. It is important for the therapist to explicitly explain this to make racism-relevant exposures safe for the client. Examples of appropriate exposures may include interactions with European American individuals who are more likely to be “safe” to talk to, such as friendly congregants at the client’s church. A bad exposure may be having the client attend a White supremacist meeting. By exposing the client to situations entailing overt racism, clinicians risk evoking feelings of anger or overwhelming anxiety, which can undermine treatment. Having clients experience such emotions at the wrong time is an example of erroneous flooding which is potentially detrimental to exposure therapy [80] and provides an example of the importance of adhering to the gradual hierarchical exposure process.

### 2.5. Imaginal Exposure

Imaginal exposures involve the client recounting memories of traumatic events both during and between therapy sessions. Imaginal exposures allow for habituation to occur, which refers to a decrease in anxiety without the use of the unhelpful safety and avoidance behaviors [81]. Similar to *in vivo* exposures, imaginal exposures aim to alter erroneous thought patterns and fears associated with the trauma which persist through avoidance behaviors. Consistent application of imaginal exposures will provide clients with an increased sense of self-efficacy and a decrease in symptoms.

Revisiting traumatic events through imaginal exposures are an excellent way for clients to see that while the event itself was dangerous, thoughts about the event recollected in a safe place are not dangerous. These exposures allow individuals to discern between actual threats and cognitive representations of threats, as well as similar events, situations, and people who they may associate with a particular trauma [78].

During imaginal exposures, the therapist plays a supportive role while guiding the client’s recounting of the experiences. Clients gain ease with sharing because of the safe therapeutic environment provided by the therapist. The most vital aspect is the client’s ability to speak freely and openly about uncomfortable feelings without judgment or criticism. For example, if a client reports a racist experience, the therapist should not later question the client as to whether or not it was actually motivated by racism or if the client elicited the event in some way. Rather the therapist should focus on how the event makes the client feel and meaning of the experience to the client.

When conducting imaginal exposures with African Americans that have experienced racism-related trauma, the therapist must have an appropriate comfort-level discussing race and should avoid minimizing race-related concerns, especially when discussing traumatic events. A useful way for therapists to make clients comfortable is to take a genuine interest in the client’s experience by taking a sufficient amount of time to explore the race-related trauma while conveying an authentic sense of care toward the client and their situation.

Therapists should ask directly about thoughts and feelings related to minority status in the face of the trauma. If these emotions are attached to racist experiences that occurred during a trauma, one may subsequently attach negative emotions to their minority status. Specific ways of assessing the impact race had on a particular trauma may be asking how being a minority in that moment made them feel, which may uncover feelings of inferiority or shame due to race. Not addressing these feelings may undermine treatment, as focusing on the impact of the trauma itself tends to be the main focus of PE therapy, and not the role race may have played. Feeling inferior or shameful due to one’s race may fuel harmful cognitions attached to the trauma. Once these thoughts and feelings are uncovered, the therapist can explore them with the client during the processing phase. The same direct questioning should be used for individuals experiencing microaggressions, as these smaller instances of racism may still produce similar feelings of shame or inferiority.

There are a variety of ways that therapists can explore feelings of minority status during traumatic experiences. A useful method may be to ask about feelings during the trauma, but also assessing race-related aspects prior to, during, or following trauma exposure. Certain situations that may occur outside of the traumatic experience, such as telling a European American doctor about being raped, may be important to ask about directly, as a negative reaction from that doctor may have an impact on feelings toward the trauma. Other examples include asking clients about any racism experienced during the actual trauma, such as slurs or comments. For example, when recounting details of being rescued from a motor vehicle accident, the therapist might ask, “How did it feel to be carried by the White EMT who was speaking sharply to you?” This experience could be very upsetting to an African American client, who might say, “it felt humiliating, like he was blaming me for what happened”, indicating it is likely a relevant event to explore further. If the client says, “I really didn’t notice, I was still reeling from accident”, then it may be safe to move on. Therapists should approach this line of inquiry from a probing and exploratory position only to uncover experiences that are relevant.

### 2.6. Processing

Processing takes place right after each imaginal exposure, and assists the client in developing a new perspective about the trauma [82]. During the imaginal exposure, verbal exchanges are kept at a minimum as to allow the client to focus on revisiting the event, so processing is a time where the client can discuss any thoughts or feelings they may have experienced during the exposure in more depth. Cognitive restructuring, or the reappraisal of the events that occur during the trauma, allows the client to re-organize the experiencing of the traumatic event. This re-organization serves as a catalyst by which the client can begin to reconcile what actually occurred and the unhelpful beliefs the person holds about their role in the trauma as well. Using Socratic questioning is a good technique, and the therapist only needs to directly point out inconsistencies when the client is truly stuck. Processing aims to reduce the pain associated with the memory by expanding the context, allowing the client to take a more objective and helpful perspective on what occurred [66].

When discussing cognitive distortions and false beliefs about the traumatic event, clinicians should be comfortable discussing race. If therapists minimize or avoid racial issues, this may present to the client as a microaggression. Statements such as “I’m not sure we need to focus on race or culture to understand your symptoms” [48] are types of microaggressive statements that prevent the exploration of important issues.

In addition, strong emotions associated with experiencing discrimination and racism can lead to distorted thoughts about society and the world as a whole. Considering that society is generally more dangerous for African Americans due to racism and discrimination [21,41,83,84], it is important to acknowledge that racism may still take place, but not to the extent of their racism-related trauma. Therefore, establishing that their experience should not be generalized to all social situations also becomes a vital component of therapy. It will be essential to work through distorted cognitions by helping the client differentiate between real social limitations and distorted perceptions that have developed as a result of the trauma [6]. As therapy progresses, clients improve in their ability to talk through the experience. Along with acknowledging that racism may still take place, collaboratively planning ways to cope with racism may be extremely useful. Encouraging clients to see racist experiences not as reflections of themselves, but problematic views and actions of the individual committing racism, may be extremely beneficial in buffering retraumatization.

Asking questions about how European American individuals responded to the trauma may allow for a discussion addressing harmful cognitions. Therapists can approach racial issues by asking questions like, “Did you think the police would believe that a White man did this to you?” or “The other people at your workplace were expecting you to fail because you were African American, but is this really a failure?” or “Was it difficult to have to tell a White social worker about your assault?” If a client did experience a racism related trauma, they may be more likely to perceive interactions with European American individuals following the trauma as microaggressions, which may produce additional negative emotions apart from the actual trauma. The therapist can also use processing questions to point out individual strengths, for example, “It was very brave to press forth in a workplace where so much racism was present. What does this say about you?” Discussing the power dynamics of the traumatic event and how it related to race may assist clients in understanding why they experienced a specific feeling during the situation, and that feelings of inferiority or powerlessness due to race should not be generalized to everyday situations. It is important to normalize feelings of fear and anger following a trauma, as it is a common response and even more so if racism is a factor.

Focusing on concepts that may resonate with African American culture can improve self-efficacy surrounding a client’s ability to recover. Mentioned earlier, African Americans tend to see the therapist as a vehicle for change [22]. Along these lines, prompting African Americans to seek ways to reduce symptoms outside of traditional psychotherapy becomes relevant in PE therapy. As this population may be more likely to view therapy as only one aspect of making positive changes, suggesting culturally relevant additives to symptom reduction outside of therapy may resonate heavily with African American clients. Specific ways to incorporate culturally relevant techniques to dispel cognitive distortions may be to focus on how clients can grow from the trauma and how they can use negative experiences to help others. For example, one common Afrocentric value is overcoming adversity, so it may be particularly meaningful to emphasize ideas such as this with African American clients.

## 3. Case Examples

### 3.1. Case # 1: PE for Treatment of Racism-Related PTSD

The client, Ms. Wells (names and identifying information have been changed for case examples), was a 49 year-old African American female living with a partner, teenage daughter and partner’s daughter. She had two master’s degrees (MBA and Technology), and worked as an IT professional at a large firm making an annual salary of $85,000. At her job, she conducted international trainings in computer security, and served as a role model to other Black women at her workplace. Although she experienced stress at work due to being the only Black female in her position, she had no psychiatric history. She had also experienced many acts of racism over the years and coped well with them.

Ms. Wells was conducting training overseas and was invited to dinner with another senior co-worker, who was a former soldier in a violent military operation. After having several drinks, the coworker began to boast about torturing and killing people, while also making racist statements. In addition, he threatened the client’s life and made sexual advances toward her. Being in a foreign country during the incident, surrounded by White people, Ms. Wells felt more vulnerable and did not think local authorities would be willing or able to protect her. She spent the whole night awake in her hotel room, locked in the bathroom holding a small knife, until she was sure the perpetrator had boarded a plane back to the U.S.

After returning, the perpetrator was fired from his current position because of his actions, and Ms. Wells sought out protection from her employer. Her employers assured her that she would be safe and company security would be on alert. This put her at ease until she later learned that no precautions to protect her were actually implemented. Subsequently, the client became paranoid about her European American co-workers, and felt as if they were all “out to get her”. Since she over-performed at work, she wondered if the perpetrator traumatizing her was a premeditated attempt to punish her in collusion with other jealous higher-ups. She could not return to work, as she was afraid to leave home alone and was experiencing panic attacks and agoraphobia.

Ms. Wells was assessed and treated by one of the authors (MTW) at an expert PTSD treatment center. The client was tearful during the interview and expressed many fears about her work environment. She was advised to seek worker’s compensation for the cost of treatment. The client also expressed that she was only willing to work with a female African American therapist. Ms. Wells met criteria for severe PTSD and Major Depressive Disorder (MDD), although the MDD appeared to be secondary to the PTSD, as were her panic attacks and agoraphobia. Her symptoms were causing great difficulty, interfering with her ability to work or interact with others. The client received PE treatment for her PTSD; after completion she was no longer fearful of the memory of the trauma but was still fearful of returning to work.

Ms. Wells’ trauma was compounded by ethnoviolence and past racist experiences including microaggressions at work. Maladaptive cognitions in PTSD including self-blame, negative thoughts about the world, and negative thoughts about the self, and African Americans tend to have more negative thoughts about the world (and less self-blame) compared to European Americans [11,12]. These negative thoughts can be due to racial microaggressions experienced across the lifetime, by witnessing openly racist acts throughout the lifetime, and/or ethnoviolence committed toward an individual [5] as in the case with Ms. Wells.

Due to the specific needs of Ms. Wells, the treating psychologist consulted with a psychologist who was board certified in African-centered psychotherapy. Treatment for the client was adapted to focus on her expectations about the workplace, experience of racism, and fear that there was no safe place for her. In addition, significant time was spent attempting to improve her self-concept and feelings of competence using Afrocentric principles, such as resiliency, optimism, faith, and family support. Considering the possibility that race-based stressors may reoccur, being able to anticipate racism becomes important. Therefore the therapist worked to instill realistic expectations about co-workers and possible negative outcomes. This prepared the client to better cope with racism in the workplace, as assuring the client that no such problems will ever occur would be unrealistic and prove detrimental (retraumatizing) in the long run. Furthermore, the client and therapist explored the possibility that she may need to find another employment opportunity with a healthier environment, and they worked together to develop goals and make an “escape plan” for future career aspirations (e.g., a list of other potential jobs and a plan for transitioning).

Following the culturally-adapted PE treatment, Ms. Wells greatly improved. She was referred to the board certified African-Centered therapist for the remainder of her treatment. However, worker’s compensation personnel from her employer wanted to have her reevaluated by their selected clinicians (European Americans, males), assessed with Eurocentric measures, stop paying for treatment, and have the client return to work prematurely. These events invalidated her traumatic experience, were retraumatizing to a degree, and ultimately slowed recovery. Ms. Wells and her therapist conceptualized these experiences as institutionalized racism [85] and worked together to problem-solve and overcome the difficulties posed.

### 3.2. Case # 2: Culturally Modified PE for Prevention of PTSD

Tracy (names and identifying information have been changed for case examples) was a single 23 year-old African American female presenting to the emergency department (ED) of a university-affiliated hospital in a large southeastern urban setting immediately following a sexual assault by her African American male roommate. She subsisted on student loans and the assistance of her roommate. She was high achieving academically and reported having a strong support system comprised mostly of African American male friends from school. She was in regular contact with her family, who resided locally, but did not live with them due to conflict with her mother. At the time of treatment, she had moved into a homeless shelter and had no prior psychiatric history. She endorsed an extensive trauma history consisting of two prior acquaintance rapes, and reported that these were also perpetrated by African American males she considered friends at the time.

On the night of the assault, the Tracy awoke from sleep to her roommate sexually assaulting her. Despite repeated verbal and physical resistance, he continued the assault. She freed herself by kicking him, left the apartment and eventually exited the locked building, while the assailant followed her, and verbally threatened to injure her. She feared he would kill her. Tracy fled to a nearby train station and approached onsite security, who contacted the police. Police officers arrived on the scene and the client assisted them to identify the assailant at their residence. The police then drove her directly to the ED, where she was administered medical treatment and a rape kit. Once medically cleared (6 h post assault), the client was invited to participate in a PTSD prevention study being conducted at the hospital [86]. The research study and participant rights were explained in detail and the client expressed interest in participating.

Tracy was evaluated for study eligibility and, upon meeting criteria, voluntarily enrolled. Clinician-rated and self-report measures were then administered to the client in her examination room to evaluate her mental health history and reaction to the recent assault. An assessment of her attributions of blame and responsibility relevant to the assault revealed that she believed she shared blame between herself and the perpetrator. Tracy initially presented as tearful and was eager for help since, she reported, “this (sexual assault) happened so many other times.” She did not meet criteria for PTSD in relation to either prior assault.

Tracy received a total of three 60-min weekly sessions of modified Prolonged Exposure therapy (m-PE) delivered in the ED by one of the authors (EM). Session 1 was delivered 8 h post assault and included a treatment rationale, psycho-education about normal reactions to trauma, 30 min of prolonged imaginal exposure to the assault, processing of trauma related themes, breathing retraining, and assigning homework. The therapist collaborated with Tracy to identify behavioral exposures to safe reminders of the assault to corrective unhelpful beliefs, self-care tasks, and helpful self-statements. These exercises were assigned for daily homework along with breathing retraining. Tracy also received a session recording and was asked to listen to the imaginal exposure daily to facilitate processing of the trauma memory. Sessions 2 and 3 included the same treatment components along with homework review.

Themes processed in Session 1 included feelings of self-blame, betrayal and anger toward the accused, whom she had confided in about her prior assaults. Tracy identified speaking to her case worker at the shelter and spending time in public with safe male friends (fellow college students) as a useful exposure to disconfirm her posttrauma belief that African American men are generally dangerous and untrustworthy and should be avoided. As an African American, Tracy expressed extreme distress over feeling “repulsed” by African American men, and believing negative stereotypes of them, labeling this “internalized racism” [87]. The therapist addressed this by differentiating internalized racism from the generalization of fear activation normally resulting from trauma that actually explained the client’s response. The therapist walked through feelings of distress and assisted Tracy in examining cognitions related to the “repulsed” feelings triggered by African American males. Negative self-statements about African Americans attached to the “repulsed” feelings were examined and appropriately attributed to assaultive behaviors related to the trauma. The therapist assisted Tracy in differentiating the fear activation associated with the trauma. It was explained that people use common factors to help them identify future attackers to avoid harm and in this race and gender were the most salient. The therapist clarified that the latter accurately explained her response since it occurred posttrauma in relation to stimuli that reminded her of her perpetrator, and she had no prior history of stereotyping or fearing African American men. Tracy reported that she experienced great relief from this discussion and felt strengthened by it to engage in the exposure exercises she selected for homework. Treating herself to a meal outside the shelter, listening to soothing music, attending college classes, and reminding herself of her goal to complete college were identified as helpful self-care tasks and statements to practice during the week.

In Session 2, Tracy reported complying with the homework but had difficulty tolerating the audio recording. A new theme of helplessness emerged and was reframed in processing as a sense of triumph for escaping from a dangerous situation. Given that Tracy had been assaulted during overnight stays with male friends in the past (and in this case a roommate), the therapist worked with her to establish safety rules around men. The theme of learning from the traumatic event in order to feel safe moving forward with her life, without stereotyping African American men or engaging in self-blame for the assaults, was discussed. Homework remained the same with the addition of a self-coaching exercise where she would process through the re-experiencing, removing herself from the situation, and effectively assisting law enforcement in identifying the perpetrator.

She complied with homework again in Session 3 and reported relief that feelings of “fear” and “repulsion” around safe male friends were diminishing. In fact, she was moved by the support she received from two male friends in whom she confided, with whom she discussed ways she could better distinguish trustworthy from untrustworthy males. Imaginal exposure focused on two hot spots, including awakening to sexual penetration by the perpetrator and returning to the residence with police investigators. Following imaginal exposure, Tracy identified “it’s not my fault” and “I am strong” as statements that would assist her to move forward from the trauma. She was encouraged to continue prioritizing self-care, which included spending time with her male friends at college and focusing on her career goals.

After being victimized a third time, Tracy needed to identify a way to feel safe again in her life. As commonly occurs in the immediate aftermath of a trauma, Tracy erroneously associated specific features of her assailant (African American, male) with dangers that are not objectively dangerous (African American, male). This resulted in distress over what she erroneously perceived to be internalized racism. The therapist contextualized Tracy’s erroneous generalization as a normal reaction to trauma that she was vulnerable to developing as an individual reared in a culture that perpetuates negative stereotypes of African American men [41]. This allowed the client to reframe and face her fears, which was necessary to access her support system (trustworthy friends who were also male and African American). It was also important for the therapist to work with Tracy on developing competency for safety and protection without resorting to stereotype-driven avoidance.

Note that this particular case example does not necessarily outline a reaction to racism-related trauma and subsequent treatment, but illustrates ways to construct exposures based on the race of the perpetrator when salient, and how to work with clients who are struggling with racial issues related to the trauma.

## 4. Discussion

### 4.1. Assessment Issues

PTSD resulting from or exacerbated by race-based trauma may be overlooked by standardized assessments, creating racial disparities in both diagnosis and treatment. While psychometric differences across race may have been noted in some assessments, most have not been validated with primarily African American samples [88] (e.g., CAPS, PCL, *etc.*). This leaves an important gap in our understanding of trauma salient to minority populations, potentially creating discrepancies in treatment and recovery. Differences to consider may include the race and ethnicity of clinician/client, understanding of race-related components, and differences in trauma cognitions across race, among others. Furthermore, given that prior events of racism may sensitize individuals to PTSD from any other type of subsequent trauma [6,51], future research should focus on validating assessments and diagnostic interviews with African American samples to gain a better understanding of the phenomenology of PTSD specific to this population.

### 4.2. Treatment Issues

The therapist must be both knowledgeable, and open to discussions concerning race and racism to fully understand the client’s experiences. For African Americans, racism-related traumatic experiences may be difficult to discuss with European American therapists, therefore clinicians must take initiative to validate these experiences and incorporate them into treatment. PE enables clients to revisit potentially racist experiences and later reprocess their meaning, an opportunity unique to PE. Acknowledgement of such experiences by the therapist will improve upon many of the barriers to treatment present for African Americans. However it is important for therapists to adhere to the PE protocol, and only incorporate the suggested adaptations when warranted. Furthermore, these adaptations may be useful if used in conjunction with other interventions other than PE, but future research is needed to assess potential efficacy.

Typically, racism qualifies as trauma only if an individual meets criteria for PTSD concerning a specific racist event. African Americans may have experienced a triggering event that resulted in trauma reactions, but have also been dealing with an aggregate of racist experiences across the lifespan. Therefore, in addition to focusing on a single discrete event, it is important to examine the history of discrimination for African Americans in therapy.

### 4.3. Limitations and Future Directions

Although there is a dire need for PTSD treatment adapted to assess racism-related trauma in African Americans, scant research has been done to assess its efficacy, subsequently limiting the clinical applicability of our PE adaptations. Specifically, research is needed to evaluate where racism-related trauma fits within the DSM-5 PTSD criteria. Race-based trauma as an option would alert clinicians to assess for this type of PTSD. The current article noted some ways in which this race-based trauma may fit within the DSM-5, but this is not yet based on sufficient empirical data.

Based on our collective clinical experience, as illustrated by the case studies included herein, we believe the suggested adaptations will improve therapeutic outcomes; however, research is needed to further substantiate this. The suggested modifications to the Standardized Trauma Interview and the PE protocol could be considered a new approach, requiring validation. Along with this, many suggestions throughout the paper concerning ways to incorporate culturally relevant principles (*i.e.*, faith based or Afrocentric approaches) into PE therapy and improving cultural sensitivity, have yet been shown to enhance therapeutic outcomes for PTSD in African Americans in randomized controlled trials (RCT). These are important areas for future research. Other therapeutic approaches may also be of value for African Americans suffering from PTSD, and these should be investigated as well.

## 5. Conclusions

This article provided an overview of PE therapy for PTSD, incorporating issues relevant to African Americans, and examples of how to adapt PE to incorporate instances of racism-related stress. African Americans face many barriers to PTSD treatment, and may have difficulties discussing certain aspects of trauma because of concerns about being misunderstood or invalidated due to race. A culturally-sensitive approach to PTSD treatment will facilitate the delivery of an effective form of therapy to a population that is disproportionally impacted by trauma. Therapists must also be well-informed of race-based trauma and unabashed by authentic discussions concerning race [42].
